# Patient, Caregiver, and Physician Barriers to Home-Based Palliative Care: Findings From a Terminated Study

**DOI:** 10.1093/geroni/igab046.633

**Published:** 2021-12-17

**Authors:** Susan Enguidanos, Stephanie Wladkowski

## Abstract

Despite two decades of palliative care services, there remains numerous barriers to patient and caregiver use of palliative care. For many years, policymakers believed lack of funding for palliative care was the primary obstacle to accessing palliative care services. In 2017, we undertook a randomized controlled trial to test the effectiveness of a home-based palliative care (HBPC) program within accountable care organizations and in partnership with an insurance company that covered the cost of HBPC. After 20 months, we had recruited just 28 patients. This symposium will: (1) describe outcomes from various approaches undertaken to engage primary care physicians and recruit patients and their caregivers into this trial; (2) present barriers to HBPC referral identified from a qualitative study of primary care physicians; (3) present findings from a qualitative study of patient- and caregiver-identified barriers to HBPC; (4) describe physician and patient barriers to research participation; and (5) discuss implications of these findings for researchers and healthcare providers. Information presented in this symposium will inform researchers and policy makers about challenges and facilitators to recruiting patients, caregivers, and physicians to participate in research studies as well as inform healthcare practitioners of potential obstacles to increasing patient access to HBPC.

